# Adsorption of Methyl Red and Methylene Blue on Carbon Bioadsorbents Obtained from Biogas Plant Waste Materials

**DOI:** 10.3390/molecules28186712

**Published:** 2023-09-20

**Authors:** Robert Wolski, Aleksandra Bazan-Wozniak, Robert Pietrzak

**Affiliations:** Department of Applied Chemistry, Faculty of Chemistry, Adam Mickiewicz University in Poznań, Uniwersytetu Poznańskiego 8, 61-614 Poznan, Poland; robert.wolski@amu.edu.pl (R.W.); aleksandra.bazan@amu.edu.pl (A.B.-W.)

**Keywords:** biocarbons, physical activation, surface chemistry, organic dyes, kinetic study

## Abstract

In this study, biocarbon was obtained from the waste material corn digest. Carbon adsorbents were obtained by physical activation of the precursor with CO_2_. Detailed physicochemical characterization of the biocarbon was carried out using low-temperature nitrogen adsorption/desorption, Boehm titration, zero-charge point (pH_pzc_) and iodine number. In addition, the sorption capacity of the biocarbon agents towards an aqueous solution of methylene blue and methyl red was determined, and the kinetics of the adsorption process were determined. The biocarbon adsorbents were characterized by an average developed specific surface area covering the range from 320 to 616 m^2^/g. The sorption capacity of the biocarbon adsorbents against methylene blue ranged from 40 mg/g to 146 mg/g, and for methyl red it covered the range from 31 mg/g to 113 mg/g. It was shown that the efficiency of organic dye removal by the obtained biocarbons depends on the initial concentration of the adsorbate solution, its mass, shaking rate, adsorbent–adsorbate contact time and temperature. The results obtained from the Langmuir and Freundlich kinetic models showed that the Langmuir model is the most suitable model for describing the adsorption of the studied pollutants on biocarbon. In turn, the adsorption kinetics of dyes is described according to the pseudo-second-order model. Adsorption studies also showed that as the process temperature increases, the removal efficiency of methylene blue and methyl red increases.

## 1. Introduction

Organic dyes cause environmental pollution. Such compounds are used in many industries. The textile industry in particular is a major consumer of dyes. It is a globally prevalent industry that accounts for roughly 7% of the aggregate global trade and engages 35 million employees [1,2]. Textile plants consume large amounts of energy and significant amounts of water. Water is used in bleaching, dyeing or washing processes [3]. The textile industry discharges untreated wastewater into water bodies. They account for more than 80% of all waste generated by this industry [4]. A significant portion consists of organic dyes, which are well soluble in water, making it very difficult to remove them by standard methods [5]. The presence of organic dyes in water bodies causes a change in color, which negatively affects the perception of the landscape [6]. On the other hand, the presence of dyes decreases the penetration of light into the lower layers of water which consequently causes a decrease in the rate of photosynthesis and reduces the concentration of oxygen necessary for life for aquatic flora and fauna [7]. Organic dyes can have toxic, mutagenic and carcinogenic effects on organisms [8,9,10,11]. By following trophic chains, it has been found that the concentration of dyes in organisms increases as the trophic chain lengthens, causing the phenomenon of bioaccumulation [12]. Dye-contaminated water, especially azo dyes, is used to irrigate fields in developing countries. This affects the biological degradation of the soil, as these compounds negatively affect soil microorganisms, causing them to die. Furthermore, plant germination and growth are hindered [7,13,14].

It is necessary to treat wastewater containing harmful dyes before it enters the environment to reduce or completely eliminate harmful effects on water, animals and humans [15,16]. Several decades ago, no special attention was paid to the discharge of wastewater containing organic dyes into the environment. Observed environmental as well as health problems prompted attention in the mid-1990s to issues related to the treatment of wastewater from the textile industry [17]. Current dye removal methods can be divided into three categories: physical, chemical and biological [14]. Biological methods are widespread, are a combination of aerobic and anaerobic processes, and are carried out before the wastewater is released into the environment. This method is inexpensive and easy to apply [18]. However, it does not completely remove dyes, and thus this type of pollution is further observed in the environment. Due to the fact that we use living organisms for purification, one disadvantage is the rate of colony growth and sensitivity to the toxicity of the dyes themselves [19]. Chemical methods use chemical compounds and the processes between them to remove dyes. These include advanced oxidation processes (AOP), electrochemical destruction, Fenton reaction dye removal, oxidation, ozonation, photochemical and ultraviolet radiation, or combinations of these methods. These methods are expensive due to the use of a large number of chemical compounds, require the construction of special facilities, and are energy-intensive [20]. During the above-mentioned processes, secondary compounds with the same or increased toxicity may be formed, which can pose additional problems during their disposal [21]. Physical methods for dye removal focus on the processes of adsorption, coagulation or flocculation, ion exchange, membrane filtration, nanofiltration or ultrafiltration, and reverse osmosis. These methods are the most commonly used because of their efficiency and easy application. Physical methods do not require as many chemical reagents as biological and chemical methods [22].

The use of activated carbon in the removal of dyes from wastewater is currently one of the most effective treatment methods compared to other adsorbents. Currently, the development of research is directed towards obtaining activated carbon from renewable resources. These include biomass, natural materials and even organic waste. The cost of obtaining activated carbons from renewable materials is in most cases lower than from non-renewable materials [23,24]. The type of raw material used, the elemental carbon content and the method of preparation determine the physicochemical and sorption properties of activated carbons [25]. Many studies indicate that activated carbon is an effective adsorbent for the removal of organic dyes [26]. In this study, the precursor for the production of biochars were the fermentation residues of corn (*Zea mays*) stalks and leaves. This precursor may be a new cheap precursor for production of carbon adsorbents, the first of all biocarbons/activated carbons. An important feature of biocarbons is that they can be effective adsorbents for various organic compounds such as methylene blue or methyl red. In addition, carbon adsorbents were synthesized using conventional heating with a pyrolysis temperature of 400 °C to investigate the feasibility of obtaining efficient adsorbents at lower temperatures.

The aim of the study was to obtain biocarbon from the waste material that was corn digest and test its adsorption capacity against an aqueous solution of methylene blue and methyl red. The biocarbon adsorbents were obtained by physical activation of the starting material in a CO_2_ atmosphere. The effect of activation temperature on the physicochemical and sorption properties of the obtained biocarbon was studied. Physicochemical (specific surface area, elemental composition, acid-base properties, pH_pzc_) and sorption (iodine, methylene blue, methyl red) properties of the obtained biocarbons were studied. Various experimental parameters such as the mass of the biocarbon, the shaking rate of the adsorbent/adsorbate system, the contact time and the concentration of the dye affecting its removal efficiency were investigated. To understand the adsorption process, two isothermal models were analyzed: the Langmuir and the Freundlich. Adsorption kinetics was studied by fitting experimental data to pseudo-first-order and pseudo-second-order models. Thermodynamic studies of the sorption process were also carried out to understand the practical suitability of the adsorbent for treating wastewater from liquid contaminants.

## 2. Results and Discussion

### 2.1. Physicochemical, Acid-Base Properties of the Precursor, Pyrolysis Product and Biocarbons Obtained

Based on the data presented in Table 1, it can be concluded that the raw material used has a low elemental carbon content in its structure (43.2 wt.%.), as well as a high amount of oxygen determined at more than 45 wt.%. In addition, a high sulfur content was observed. The data presented in Table 1 show that pyrolysis and activation processes affect the elemental composition of the obtained biomaterials. It was observed that pyrolysis of the starting material leads to biochar with nearly twice the elemental carbon content of the starting material. In addition, a decrease in the amount of hydrogen and oxygen was observed in the P sample, as well as an increase in the content of sulfur and nitrogen relative to the precursor. The content of C^daf^ in the structure of the obtained biocarbons ranges from 59.4 to 74.6 wt.%. Further analysis of the results summarized in Table 1 also allows us to conclude that raising the activation temperature by 100 °C contributes to a decrease in the proportion of carbon, hydrogen and nitrogen compared to the PA6 sample. On the other hand, analysis of the sulfur and oxygen content allows us to conclude that increasing the activation temperature results in an increase in the content of these heteroatoms. Table 1 also shows the performance of pyrolysis and activation processes. It is known that the efficiency of biocarbon synthesis consists of a number of factors: the type of precursor and activation agent, and the time and temperature of the pyrolysis and activation process. The efficiency of synthesis turned out to be highest in the case of pyrolysis, at 59.8%.

The acid-base properties of the precursor and biocarbons were studied and are shown in Table 2. Using the Boehm titration method, the amount of oxygenic acidic and basic functional groups was determined. In addition, the pH of the aqueous extracts of the starting material and the obtained bioadsorbents was measured to confirm the results obtained. The starting material shows an acidic character of the surface (pH 4.29) because a significant predominance of acidic (1.35 mmol/g) over basic (0.44 mmol/g) groupings was recorded for this material. It was observed that regardless of the activation temperature, PA6 and PA7 adsorbents have a less acidic surface character compared to the starting material. Increasing the activation temperature causes a decrease in acidic character groupings and an increase in basic groupings in the structure of PA6 and PA7 samples. The richer surface chemistry is shown by the PA6 adsorbent, which has 0.92 mmol/g of acidic groupings and 0.31 mmol/g of basic groupings. In contrast, 0.69 mmol/g of acidic groupings and 0.42 mmol/g of basic groupings were recorded for the PA7 sample.

**Figure 1:**
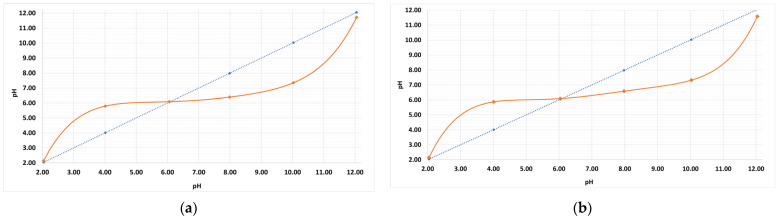
Determination of pH_pzc_ values for biocarbon PA6 (**a**) and PA7 (**b**).

pH_pzc_ helps select the optimal pH value for adsorption testing. The pH_pzc_ value means that at this value the surface charge of the adsorbent is zero. A pH value of the solution (system) higher than pH_pzc_ means that the surface of the biocarbon is negatively charged and can interact with the positively charged adsorbate (dye). On the other hand, at a pH lower than pH_pzc_, the surface of the adsorbent is positively charged which allows it to effectively interact with the negatively charged adsorbate. The pH_pzc_ values of PA6 and PA7 were determined using the drift method (Figure 1). For PA6 the pH_pzc_ is 6.10, and for PA7 it is 6.20. The results indicate a slightly acidic character of the surface of the samples, which is characteristic of biocarbons obtained by physical activation with carbon dioxide.

**Figure 2:**
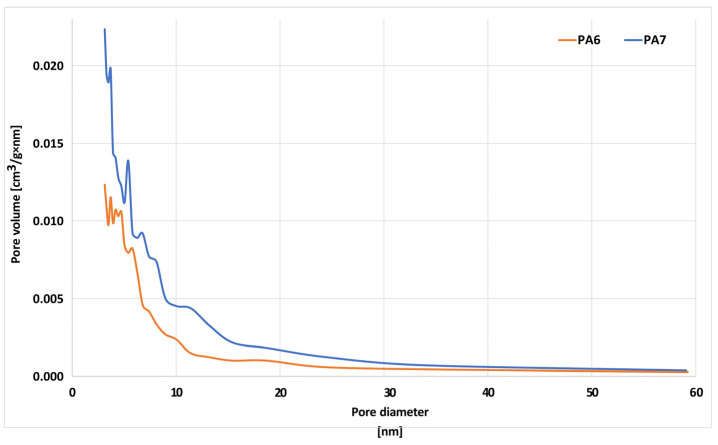
Pore size distribution of the biocarbons.

The textural parameters of the biocarbons are shown in Table 3. The PA6 sample was found to have a surface area of 320 m^2^/g, while the PA7 biocarbon had a surface area of 616 m^2^/g. The poorly developed specific surface area of the obtained bioadsorbents may be due to the fact that the precursor contained significant amounts of mineral matter in its structure, which blocked/clogged the pores, thus preventing an effective reaction between the precursor and activator (carbon dioxide). Further analysis of the data summarized in Table 3 allows us to conclude that an increase in activation temperature by 100 °C results in an increase in surface area of nearly 300 m^2^/g. It is possible that the temperature fluctuations during the pyrolysis and activation stages are sufficient to boost the porosity of the pyrolysis product, resulting in an increase in surface area. Analysis of the data also allows us to conclude that the structure of the obtained biocarbons is dominated by micropores and small mesopores (Figure 2, Table 3), as evidenced by the values of the average pore diameter which amounted to, for the PA6 sample, 3.96 nm, while for PA7 it was 3.20 nm. On the other hand, taking into account the values of pore volume and micropore volume, it can be concluded that more micropores are contained in the structure of biocarbon PA7.

**Figure 3:**
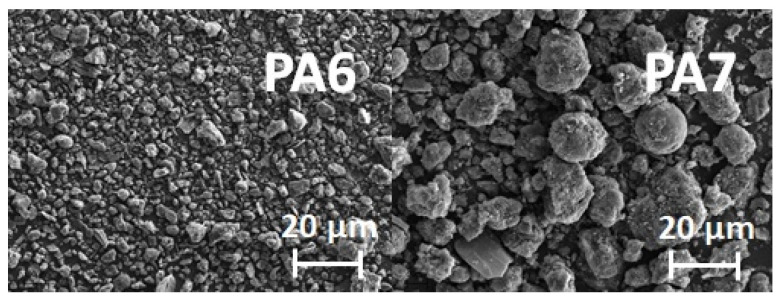
SEM images of samples PA6 and PA7.

SEM images of the PA6 and PA7 samples are presented in Figure 3. Differences in pore size, shape and number are observed between the samples. The presence of ash may account for the brighter fragments found in the bioadsorbents.

**Figure 4:**
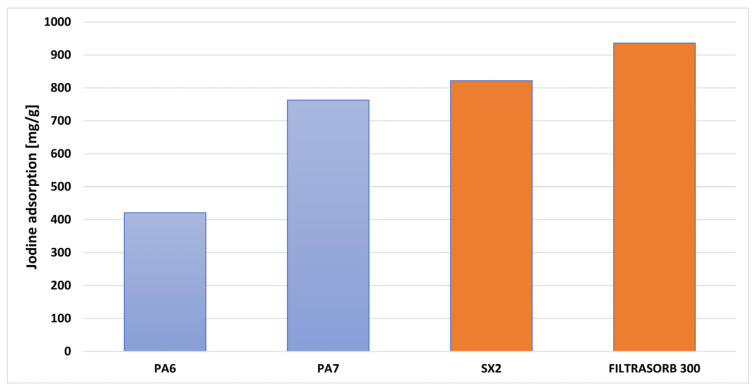
Iodine number of sample PA6 and PA7 and commercial products.

Analysis of the data presented in Figure 4 allows us to conclude that the obtained biocarbons show the ability to remove inorganic contaminants with sizes close to 1 nm. Figure 4 shows the iodine numbers of biocarbons PA6 and PA7 and two commercial carbons: SX2 (obtained from peat by Norit Activated Carbon, Zaandam, The Netherlands) and Filtrasorb 300 (obtained from bituminous coal by Calgon Carbon Corporation, Pittsburgh, PA, USA).

Based on the results, it can be concluded that the efficiency of iodine removal depends on the activation temperature. The most effective adsorbent turned out to be biocarbon PA7. The sorption capacity of this carbon was 763 mg/g. In contrast, the PA6 sample was able to adsorb 421 mg/g of iodine. The higher sorption capacity of biocarbon PA7 is probably a consequence of its better developed specific surface area and microporous structure, which favors the sorption of impurities with small particle sizes. The results obtained for the PA7 sample were slightly inferior to the commercial activated carbons: SX2 was 822 mg/g and Filtrasorb 300 was 936 mg/g.

### 2.2. Adsorption Study

**Figure 5:**
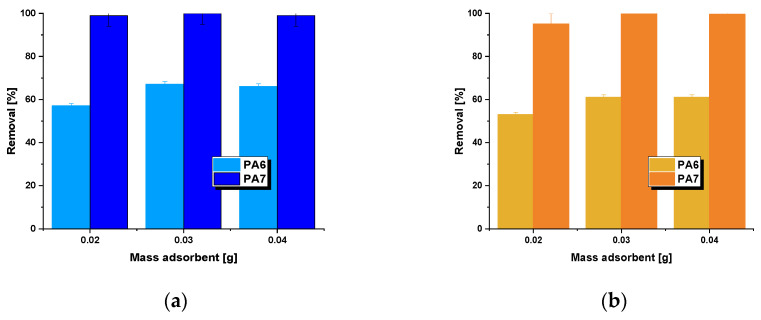
Effect of adsorption efficiency on the adsorption of methylene blue (**a**)/methyl red (**b**) on biocarbons. Methylene blue adsorption conditions: initial concentration of dye solution = 50 mg/L, volume of solution = 50 mL, shaking speed = 250 rpm/min, pH = 5.95, temperature = 22 ± 1 °C). Methyl red adsorption conditions: initial concentration of dye solution = 30 mg/L, volume of solution = 50 mL, shaking speed = 250 rpm/min, pH = 7.30, temperature = 22 ± 1 °C.

At the beginning of the adsorption study, the effect of biocarbon mass on the obtained sorption capacities towards aqueous solutions of methylene blue (Figure 5a) and methyl red (Figure 5b) was determined. Data analysis indicated that the percentage removal of the tested pollutants increased with an increase in the mass of the bioadsorbent. Increasing the mass of samples PA6 and PA7 resulted in an increased contact surface area between the adsorbent and adsorbate, and consequently, an increased number of active sites capable of adsorbing the tested pollutants [27]. Further analysis of the data presented in Figure 4 also revealed that the maximum removal efficiency of aqueous solutions of methylene blue and methyl red was achieved for biochar PA7 at a mass of 30 mg. Conversely, when a higher mass of adsorbent (40 mg) was used, no significant changes were observed.

**Figure 6:**
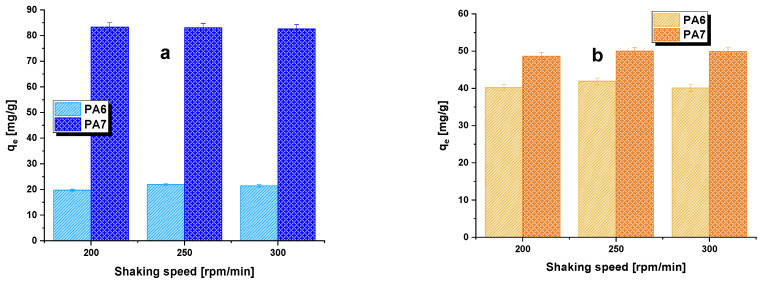
Effect of shaking speed on the adsorption of methylene blue (**a**)/methyl red (**b**) on biocarbons. Methylene blue adsorption conditions: adsorbent mass = 30 mg, initial concentration of dye solution = 50 mg/L, volume of solution = 50 mL, pH = 5.95, temperature = 22 ± 1 °C. Methyl red adsorption conditions: adsorbent mass = 30 mg, initial concentration of dye solution = 30 mg/L, volume of solution = 50 mL, pH = 7.30, temperature = 22 ± 1 °C.

The influence of shaking speed on the bioadsorbents was investigated within the range of 200 to 300 rpm/min. The sorption capacity results obtained at different shaking speeds for biochars PA6 and PA7 are presented in Figure 6. As shown in Figure 6, both biochars exhibited the same trend. The sorption capacity towards aqueous solutions of methylene blue/methyl red increased with the increase in shaking speed from 200 to 250 rpm/min and then remained essentially constant. When the samples were shaken within the range of 200–250 rpm/min, the biochar particles moved rapidly in the solution, which increased the concentration of the dye near the adsorbent surface, likely reaching a level close to the total concentration [28]. However, when the shaking speed was higher (300 rpm/min), the diffusion rate decreased. Therefore, further adsorption studies were conducted at a shaking speed of 250 rpm/min and a bioadsorbent mass of 30 mg.

In the next stage of the adsorption study, the experimental sorption capacities of the bioadsorbents for the analyzed organic dyes were determined (Table 4). Sample PA6 was able to adsorb 40 mg of methylene blue and 31 mg of methyl red. On the other hand, the experimental sorption capacity of biochar PA7 for methylene blue was 146 mg/g, and for methyl red it was 113 mg/g. Lower sorption capacities for methyl red were observed for both bioadsorbents. Furthermore, it can be concluded that the removal efficiency of the tested pollutants may be related to the textural parameters of samples PA6 and PA7. The significantly higher sorption capacities for biochar PA7 are attributed to its better-developed specific surface area compared to the biochar activated at a temperature 100 °C lower.

The experimental studies allowed for the determination of characteristic parameters for two models: Langmuir and Freundlich. Analyzing the maximum sorption capacities calculated for the Langmuir model, it can be observed that they were very close to the experimental values, q_e_. The experimentally determined sorption capacities for methylene blue on the investigated biochars were 1 mg lower than the q_max_ values. On the other hand, for methyl red adsorption, the difference between the obtained capacities was 2 mg. Furthermore, by examining the correlation coefficient values, R^2^, it can be concluded that the Langmuir model provided a better fit for both organic dyes. Therefore, it can be inferred that the formation of a monolayer adsorption occurs on the surfaces of samples PA6 and PA7 [29]. Another parameter indicating whether the adsorption process of the tested pollutants proceeds favorably is the dimensionless separation factor. The values of the R_L_ parameter for both treatments (regardless of the type of dye removed) fall within the range of 0 to 1, indicating that the adsorption process conditions were favorable [30]. The last parameter in the Langmuir model is the equilibrium constant K_L_. A higher value of this parameter indicates stronger interactions between the adsorbent and adsorbate. Therefore, considering the values of the K_L_ constant presented in Table 4, it can be inferred that the binding between methylene blue/methyl red and the biochar surface is stronger for sample PA6, as higher K_L_ values were obtained for this adsorbent [1]. In the case of the Freundlich model, lower values of the correlation coefficient R^2^ were obtained. However, considering the values presented in Table 4, it can be observed that for the adsorption of the aqueous solution of methylene blue on biochar PA7, an R^2^ value of 0.990 was obtained. This suggests that a multilayer adsorption process may also occur for this sample. Based on the parameter n determined for the Freundlich model, it can be determined whether the sorption has a physical character when n > 1, a chemical character when n < 1, or a linear character when n = 0 [31]. For the investigated samples, the value of n falls within a range greater than 1, indicating that the adsorption of methylene blue/methyl red on the obtained bioadsorbents has a physical nature. Furthermore, the closer the value of the heterogeneity factor 1/n is to zero, the greater the affinity of the adsorbate to the adsorbent. In the case of the investigated bioadsorbents, this value ranges from 0.132 to 0.330, indicating a high heterogeneity of their surfaces.

**Figure 7:**
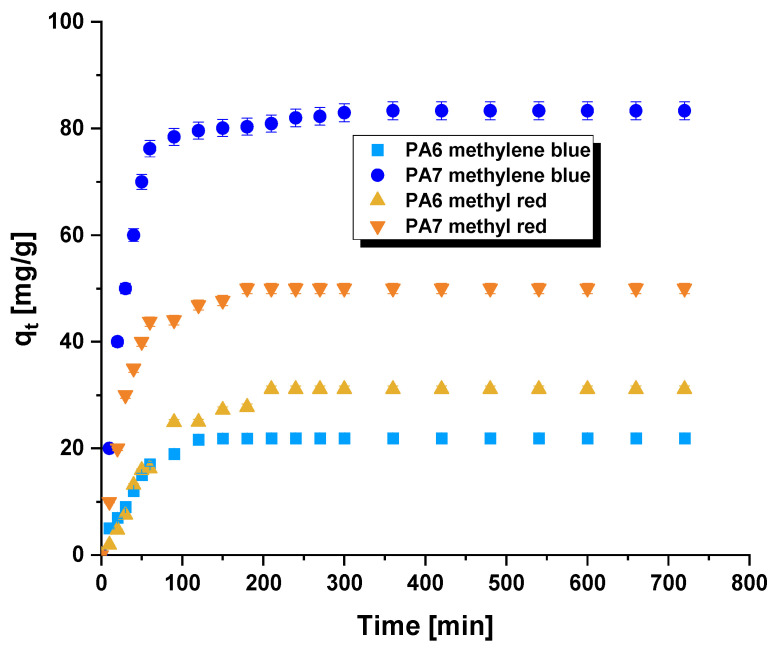
Effect of contact time on the adsorption of methylene blue/methyl red on the biocarbons. Methylene blue adsorption conditions: adsorbent mass = 30 mg, initial concentration of dye solution = 50 mg/L, volume of solution = 50 mL, shaking speed = 250 rpm/min, pH = 5.95, temperature = 22 ± 1 °C. Methyl red adsorption condition s: adsorbent mass = 30 mg, initial concentration of dye solution = 30 mg/L, volume of solution = 50 mL, shaking speed = 250 rpm/min, pH = 7.30, temperature = 22 ± 1 °C.

Figure 7 presents the adsorption isotherms of methylene blue/methyl red on the obtained bioadsorbents. Based on the shape of the isotherms, it can be observed that the amount of adsorbed organic dye significantly increases with the duration of the process. This is due to the fact that at the beginning of the adsorption process, the adsorption of the dye on the surface of samples PA6 and PA7 occurs randomly. However, as the process continues, the number of available active sites decreases (gradual saturation of active sites with dye molecules), and these sites become less accessible for dye molecules due to repulsive interactions between the adsorbed dye and the dye molecules present in the solution [30]. Analyzing the data presented in Figure 7, it can be noted that the equilibrium state was achieved fastest, within 120 min, during the adsorption of the aqueous solution of methylene blue on biochar PA6. It is also worth emphasizing that the equilibrium adsorption process was relatively fast in the other three cases as well, which is highly significant from an economic point of view.

The experimental data of methylene blue/methyl red adsorption on the surface of biochar were interpreted using pseudo-first-order and pseudo-second-order kinetic models. The parameter values for these models are presented in Table 5. Based on the correlation coefficients R^2^, it can be concluded that the pseudo-second-order model provided the best fit to the experimental data. This model is commonly used to describe chemisorption-controlled adsorption processes, where there is electron sharing or exchange between the dye (its functional groups) and the functional groups present on the surface of the synthesized adsorbents [32]. Furthermore, for all four adsorption processes, no significant differences were observed between the calculated amount of adsorbed dye (q_e,cal_) and the experimentally obtained amount (q_t_) at equilibrium.

The thermodynamics of the process is another key aspect that provides information on whether the interaction between the guest and host occurs through physisorption or chemisorption. According to the results presented in Table 5, the efficiency of removing aqueous solutions of organic dyes increased with increasing temperature. Raising the temperature of the reaction system enhances the mobility of the organic dye molecules. As a result, they can engage with the biochar surface more swiftly and effectively whilst infiltrating the adsorbent pores. However, the observed increase was slight. The largest influence of this parameter was observed in the adsorption of methyl blue on PA7 biochar (increase of 29 mg/g), while the smallest was during the adsorption of methyl red on the bioadsorbent obtained through physical activation of the starting material at 600 °C (increase of 10 mg/g). The diffusion of methylene blue/methyl red molecules is a temperature-controlled process, which is why changes in sorption capacities were observed. The increase in temperature facilitated the rapid diffusion of dye molecules into the pores of the biochar. Thermodynamic studies allowed the determination of parameters such as the change in Gibbs free energy, enthalpy and entropy (Table 6). The negative values of the ∆G^0^ parameter indicate that the process occurred spontaneously. The most negative values for Gibbs free energy were obtained at 338 K, confirming that the adsorption of methylene blue/methyl red was most favorable at this temperature. On the other hand, the positive values for enthalpy and entropy indicate that the investigated adsorption processes are endothermic [27]. The results of the ΔH^0^ parameter range from 6.88 to 12.21 kJ/mol, indicating a physisorption process. The ΔS^0^ parameter ranges from 40.11 to 63.61 kJ/mol. Positive entropy values indicate increased randomness at the interface between the biochar and the solution of methylene blue/methyl red, which increases the effectiveness of removing the studied pollutants. The higher the value of ∆S^0^, the more efficient the process occurs [33]. Therefore, considering the results presented in Table 6, it can be concluded that the most efficient adsorption process occurs for methylene blue and the PA7 sample.

**Figure 8:**
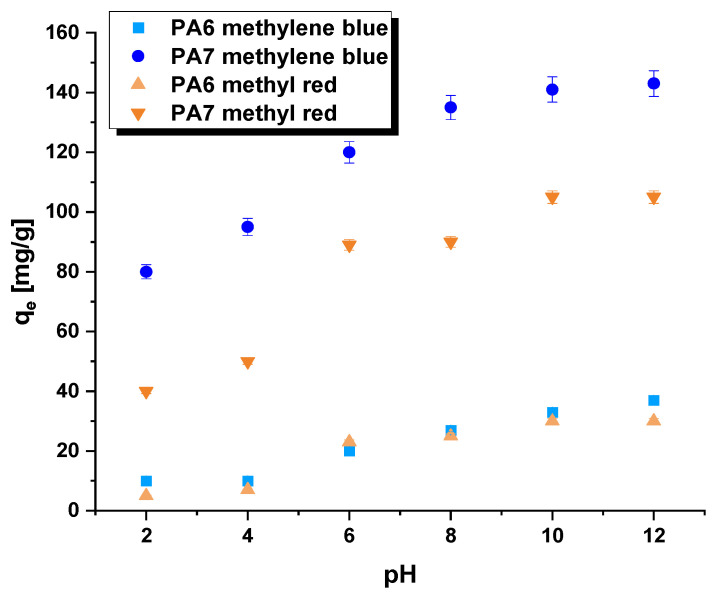
Effect of pH on the adsorption of methylene blue and methyl red on bioadsorbents. Adsorption conditions: adsorbent mass = 30 mg, initial concentration of methylene blue solution = 50 mg/L, initial concentration of methyl red solution = 50 mg/L, volume of solution = 50 mL, shaking speed = 250 rpm/min, temperature = 22 ± 1 °C.

It is expected that the pH of aqueous dye solutions will significantly impact their adsorption efficiency. During the process of adsorbing methylene blue onto samples PA6 and PA7, an increase in sorption capacity was observed with an increase in the pH of the adsorbate. In contrast, a decrease in sorption capacity was noticed during the adsorption of methyl red (Figure 8). This distinction can be explained by the structure of these dyes; methylene blue is an organic dye with a cationic charge, whilst methyl red is an anionic dye. The chosen dyes in this research are composed of diverse functional groups and aromatic rings. These functional groups have the potential to engage in π–π, hydrogen bonding, and electrostatic interactions with activated carbons. Moreover, there is a possibility of π–π interactions occurring between the dye samples and aromatic rings. Comparable interactions have been documented and discussed in existing literature [33].

**Figure 9:**
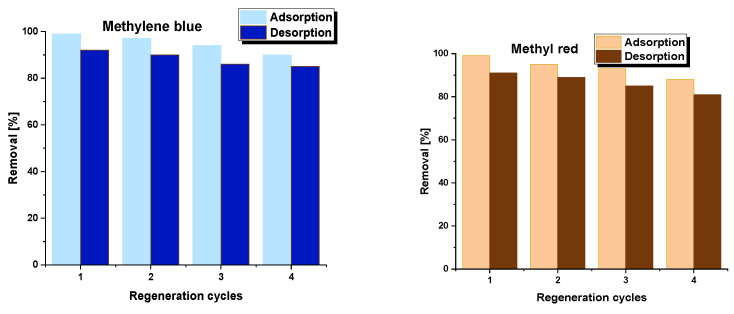
Reusability of PA7 sample. Methylene blue adsorption conditions: adsorbent mass = 30 mg, initial concentration of dye solution = 50 mg/L, volume of solution = 50 mL, shaking speed = 250 rpm/min, pH = 5.95, temperature = 22 ± 1 °C. Methyl red adsorption conditions: adsorbent mass = 30 mg, initial concentration of dye solution = 30 mg/L, volume of solution = 50 mL, shaking speed = 250 rpm/min, pH = 7.30, temperature = 22 ± 1 °C.

Regeneration is a crucial factor in determining the lifespan of adsorbents, indicating the maximum number of times they can be used in an adsorption process without losing their initial adsorption capacity. This study investigates this feature for the PA7 sample (Figure 9) using solutions of methyl red (30 mg/L) and methylene blue (50 mg/L). After four adsorption cycles, the ability to remove methylene blue remained at 90%, whereas methyl red efficiency was 88%. In contrast, the desorption efficiency for methyl red after four cycles was 81%, whereas for methylene blue, it was 83%. The biocarbon is stable and has the potential for repeated use without losing its affinity for the selected analytes.

In the final stage of the study, the obtained results were compared with the sorption capacities of adsorbents described in the literature. Table 7 presents the maximum sorption capacities (q_max_) for methylene blue and methyl red. It can be observed that the PA7 biochar, especially, exhibits high efficiency in removing methylene blue and has an advantage over other adsorbents. The PA7 sample shows significantly higher sorption capacity for methylene blue compared to biochar obtained by oxidizing weeds using HNO_3_ [34]. The bioadsorbents presented in this study also demonstrate higher efficiency in removing methylene blue compared to microcrystalline cellulose, whose maximum adsorption capacity is 13 mg/g of adsorbent [35]. Further analysis of the data presented in Table 7 reveals that only the hydrochar from wood residues of *Pinus caribaea* [36] was able to adsorb a comparable amount of methylene blue to the PA7 sample. The q_max_ values listed in Table 7 for methyl red indicate that the obtained biochars, in addition to their ability to adsorb methylene blue, also exhibit the ability to adsorb methyl red. The sample obtained by activating the carbonized material at 600 °C shows a similar sorption capacity compared to the bark of Hopbush [37] and to white potato peel powder [38]. It is also worth noting that the second obtained biochar exhibited a significantly higher sorption capacity compared to the adsorbent obtained from coffee residues, which had a q_max_ of 77 mg/g [39].

## 3. Materials and Methods

### 3.1. Precursor and Biocarbons

**Figure 10:**
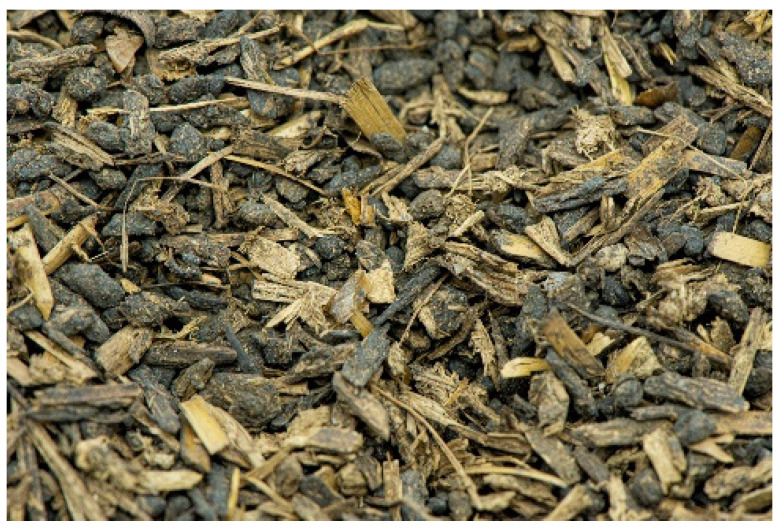
Residue from the fermentation process of corn stalks and leaves after granulation.

The precursor of the biocarbons was the fermentation residues of corn (*Zea mays*) stalks and leaves. The corn was raised and harvested in Greater Poland (western Poland). The corn fermentation residue was obtained from the biogas plant in a granulated form with different grain sizes (Figure 10). The starting material was sieved and a fraction with a gradation of 2–3 mm was taken for further study. The sieved precursor was dried at 110 °C for 24 h. The moisture content of the starting material was 10.30%.

The dried precursor was subjected to pyrolysis (P). Pyrolysis was carried out in a horizontal furnace (Thermo Fisher Scientific Inc., Waltham, MA, USA), which was equipped with a tubular quartz reactor. The process was carried out under an atmosphere of nitrogen (technical nitrogen 4.0, Linde Gaz Poland), with a wash rate of 170 mL/min. A 25 g precursor sample was placed in a nickel boat and then heated from room temperature to 400 °C. The sample was thermostated for 60 min. After this time, the sample was left in a nitrogen atmosphere to cool.

The pyrolysis product was subjected to activation (A) with carbon (IV) oxide. A nickel boat with 15 g of biocarbon was placed in a preheated 600 °C (PA6) or 700 °C (PA7) horizontal furnace. The gas flow rate (technical CO_2_ 2.8, Linde Gaz Poland) through the tubular quartz reactor was 250 mL/min. The activation time was 45 min. The sample was then removed from the heating reaction zone and cooled to room temperature in a CO_2_ atmosphere. After activation, the samples were washed hot with a 5% (*w*/*w*) hydrochloric acid solution, followed by distilled water and dried at 110 °C.

### 3.2. Precursor/Biocarbons Characteristics

Elemental analysis was carried out for the precursor, pyrolysis product and biocarbons using a Vario EL III elemental analyzer (Elementar Analysensysteme GmbH, Langenselbold, Germany). Determination of ash content was performed in accordance with DNS ISO 1171:2002.

Textural characterization of biocarbons was performed using an ASAP 2020 sorptometer manufactured by Micrometrics Instrument Corporation (Norcross, GA, USA). The analysis was based on nitrogen adsorption–desorption isotherms measured at −196 °C. Samples were degassed at 250 °C for 24 h to remove substances adsorbed on the surface of biocarbons. BET specific surface area (S_BET_) was evaluated in the relative pressure range p/p_0_ 0.05–0.30. Total pore volume (V_t_) was calculated by converting the amount adsorbed at p/p_0_~0.99 to the volume of liquid adsorbate. The average pore diameter was calculated (D) from the equation D = 4V_t_/S_BET_. The well-known t-plot method was used to determine the volume and surface area of the micropores. SEM images were obtained using a scanning electron microscope (PHILIPS, Eindhoven, The Netherlands) in the following conditions: working distance of 14 mm, acceleration voltage of 15 kV and digital image recording by DISS.

The acid-base properties of the tested materials were determined by testing the pH of their aqueous extracts and determining the number of acid and base functional groups by Boehm titration [40].

The pH_pzc_ [41] point was determined for each biocarbon. To a series of 100 mL flasks, 40 mL of 0.1 mol/dm^3^ NaCl solution was added. Nitrogen (nitrogen 4.0, Linde Gas Poland, Poznań, Poland) was passed through the solution and the pH value was corrected with HCl and NaOH solutions. The pH_intital_ was fixed from 2 to 12 in two-unit increments. Then 0.2 g of the previously powdered biocarbons sample was added. The flasks were placed on a shaker. After 24 h, the pH_final_ value was measured. The pH_pzc_ value was obtained from graphs of the relationship between pH_inital_ and pH_final_.

The iodine number of the biocarbons was determined in accordance with PN-83/C-97555.04. A 0.2 g sample of powdered adsorbent (0.09 mm) was placed in a 100 cm^3^ flask. 4 cm^3^ of 5% (*w*/*w*) hydrochloric acid (POCH, Gliwice, Poland) was added, followed by 20 cm^3^ of 0.2 mol/dm^3^ aqueous solution of iodine (Sigma-Aldrich, Darmstadt, Germany) in potassium iodide (Sigma-Aldrich, Germany). The mixture was shaken for 4 min on a laboratory shaker (Heidolph, Schwabach, Germany) at 400 rpm, then filtered through filter paper and washed with 50 cm^3^ of distilled water. The filtrate was titrated with 0.1 mol/dm^3^ sodium thiosulfate solution (POCH, Gliwice, Poland) against starch as an indicator. Three parallel determinations were made for each biocarbon.

### 3.3. Adsorption of Methylene Blue/Methyl Red

Methylene blue and methyl red were of analytical grade, purchased from Merck (Darmstadt, Germany). In the initial stage of adsorption studies, aqueous solutions of the above-mentioned organic dyes were prepared at a concentration of 1000 mg/L. From the initial solutions of methylene blue and methyl red, working solutions were prepared in the range of concentrations from 10 to 100 mg/L for methylene blue and from 10 to 80 mg/L for methyl red. The adsorption processes were conducted using a Carry 100 Biospectrophotometer (Agilent, Santa Clara, CA, USA) at a wavelength of 665 nm for methylene blue and 443 nm for methyl red. The studies were conducted at room temperature—22 °C ± 1. The experimental sorption capacities for PA6 and PA7 biochar were determined as the arithmetic mean of two independent measurements.

The adsorption studies began by determining the influence of biochar mass on the efficiency of removing the target organic pollutants. Three sample masses were applied: 0.02 g, 0.03 g and 0.04 g. The bioadsorbents were submerged in 50 mL of a 50 mg/L solution of methylene blue or a 30 mg/L solution of methyl red. The adsorption processes were conducted using a shaker (Heidolph, Schwabach, Germany) at a shaking speed of 250 rpm/min for a duration of 8 h. After shaking, the solid samples were separated from the solution using a laboratory centrifuge (OHAUS, Parsippany, NJ, USA), and the sorption capacity of the carbon adsorbents was determined based on spectrophotometric measurements. Equations (1) and (2) were used for this purpose:

(1) $$q_{e}=\frac{(C_{0}-C_{e})\times V}{m}$$

(2) $$Removal\;[\%]=\frac{\left(C_{0}-C_{e}\right)}{m}\times 100\%$$

where C_0_ [mg/L] is the initial concentration of methylene blue/methyl red, C_e_ [mg/L] is the concentration of the dye in the equilibrium state, m [g] is the total mass of PA6/PA7 and V [L] indicates the volume of the dye solution.

The influence of biochar shaking speed on the obtained sorption capacities was also investigated. Three shaking speeds were used: 200 rpm/min, 250 rpm/min and 300 rpm/min. The adsorption was conducted for 8 h using the same organic dye solutions as in the determination of the effect of bioadsorbent mass on the adsorption process efficiency. The mass of biochar was 0.03 g.

The interactions between biochar and the organic dye were determined based on two isotherm models: Langmuir and Freundlich.

The Langmuir equation describes an adsorption model that leads to the formation of a monolayer of adsorbate molecules on the surface of the adsorbent. According to the Langmuir model, the adsorbent surface has a specific number of active sites (uniformly energetically active centers), each capable of adsorbing only one molecule of the adsorbate (e.g., dye). The binding of the adsorbate molecule to the adsorbent can have either a physical or chemical nature [42]. The Langmuir equation is given by Equation (3):

(3) $$\frac{C_{e}}{q_{e}}=\frac{1}{K_{L}\times q_{max}}+\frac{C_{e}}{q_{max}}$$

where K_L_ is the Langmuir constant [L/mg] and q_max_ is the maximum adsorption capacity of the monolayer [mg/g].

Additionally, for the Langmuir equation, the dimensionless separation factor R_L_ (4) can be determined:

(4) $$R_{L}=\frac{1}{1+K_{L}\times C_{0}}$$

The R_L_ factor determines the intensity of the adsorption process. It is commonly accepted that the adsorption process is favorable if the following condition is met: 0 < R_L_ < 1. When R_L_ = 0, the adsorption is reversible, while R_L_ = 1 indicates a linear adsorption isotherm. On the other hand, an R_L_ value above 1 indicates unfavorable conditions for the sorption of a particular substance.

The Freundlich isotherm equation is used to describe heterogeneous systems [43], which can be characterized by the heterogeneity factor—1/n. The Freundlich equation is given by Equation (5):

(5) $$lnq_{e}=lnK_{F}+\frac{1}{n}lnC_{e}$$

where K_F_ [mg/g(L/mg)^(1/n)^] is the Freundlich constant and 1/n is a constant associated with the intensity of the adsorption process.

The optimization of contact time was conducted for a 50 mL solution of methylene blue with a concentration of 50 mg/L and a 30 mg/L solution of methyl red, using a bioadsorbent mass of 0.03 g while maintaining a shaking speed of 250 rpm/min. The processes were carried out for 8 h. The conducted studies allowed for the determination of kinetic parameters characteristic of the pseudo-first-order and pseudo-second-order models [26].

The pseudo-first-order model is based on the assumption that the reaction rate is proportional to the amount of ions present in the solution at a given time (6):

(6) $$\log\left(q_{e}-q_{t}\right)=logq_{e}-\frac{k_{1}t}{2.303}$$

where k_1_ [1/min] is the rate constant of the pseudo-first-order model, q_t_ [mg/g] is the amount of adsorbed methylene blue/methyl red at time t [min].

On the other hand, the pseudo-second-order model is based on the assumption that a chemisorption process occurs between the adsorbent and the adsorbate. The linear form of this equation takes the following form (7):

(7) $$\frac{t}{q_{t}}=\frac{1}{k_{2}{q_{e}}^{2}}+\frac{t}{q_{e}}$$

where k_2_ [g/mol × min] is the second-order rate constant.

In the final stage of the study, the effect of temperature on the efficiency of organic dye removal was determined. The experiments were conducted at three different temperatures: 298 K, 318 K and 338 K. The adsorption of aqueous solutions of organic dyes on PA6 and PA7 biochar was carried out for 8 h, with a sample shaking speed of 250 rpm/min, a biochar mass of 0.03 g, and a solution of either 50 mg/L methylene blue or 30 mg/L methyl red. Thermodynamic parameters [44] ΔG^0^ (standard Gibbs free energy), ΔH^0^ (enthalpy change), and ΔS^0^ (entropy change) were calculated based on Equations (8)–(10):

(8) $$\Delta G^{0}=-RTlnK_{d}$$

(9) $$\Delta G^{0}=\Delta H^{0}-T\Delta S^{0}$$

(10) $$lnK_{d}=\frac{\Delta S^{0}}{R}-\frac{\Delta H^{0}}{RT}$$

where: ΔG^0^ is the Gibbs free energy [kJ/mol], R is the gas constant [J/mol × K], T is the temperature [K], K_d_ is the thermodynamic equilibrium constant, ΔH^0^ [J/mol] is the enthalpy change, and ΔS^0^ [J/mol × K] is the entropy change.

The correlation between the sorption characteristics of the bioadsorbent samples and the pH of the dye solution was analyzed at a pH range of 2 to 12. The pH of the water solutions containing methylene blue or methyl red were adjusted using 0.1 M NaOH/HCl solution. The PA6 and PA7 samples were immersed in a dye solution of the specific concentration and pH and agitated at 250 rpm/min for eight hours. Measurements were carried out using 30 mg of the sample and 50 mL of the dye solution with a suitable concentration (methylene blue: 50 mg/L; methyl red: 30 mg/L) and pH ranging from 2 to 12.

## 4. Conclusions

Studies have shown that waste from biogas plants after the corn straw fermentation process can be successfully used as a precursor for biocarbon production. The use of waste material makes it possible to obtain a cheap source of raw material. Such action partly solves the problem of waste management. The biocarbon obtained from corn digest showed a high ash content, about 50% by weight. This is due to the conversion of some of the organic matter into biogas during the fermentation process. Analysis of the physicochemical properties proved that after physical activation of the fermentation residues of corn (*Zea mays*) stalks and leaves, carbon materials of relatively poorly developed surface area (320–616 m^2^/g and acidic character of surface are obtained. The sorption capacities of the biocarbons studied varied from 40 to 146 mg/g towards methylene blue and from 31 to 113 mg/g for methyl red from their water solutions. The adsorption process was best described by the Langmuir model, as evidenced by the high determination coefficient (R^2^) values ranging from 0.990 to 0.990. This suggests the formation of a monolayer adsorption phenomenon on the adsorbent surface. Additionally, the pseudo-second-order model effectively represented the dye removal process, implying a chemisorption mechanism between the adsorbent and the adsorbate. The methylene blue/methyl red adsorption on the bioadsorbents studied was endothermic and spontaneous.

The comparison of sorption properties with other biocarbon obtained from waste materials turned out favorably for PA6 and PA7 formulations. The findings also emphasize the need for process optimization in the production of biocarbons, including the selection of appropriate physical and activation parameters to achieve adsorbents with more favorable textural properties.

## Figures and Tables

**Table 1:** Elemental analysis [wt.%].

Sample	Ash	C^daf 1^	H^daf^	N^daf^	S^daf^	O^diff 2^	Yield ^3^
Precursor	27.4	43.2	6.5	3.5	1.6	45.2	-
P	55.8	81.3	3.2	4.5	2.3	8.7	59.8
PA6	49.6	74.6	1.8	3.6	1.3	18.7	54.4
PA7	52.7	59.4	1.6	3.2	1.6	34.2	50.8

^1^ Dry ash-free basis. ^2^ Calculated by difference; method error ≤0.3%. ^3^ In [wt.%].

**Table 2:** Acid-base properties of precursor and biocarbons.

Sample	Acidic Group Content [mmol/g]	Basic Group Content [mmol/g]	Total Content of Surface Groups [mmol/g]	pH of Aqueous Extracts
Precursor	1.35	0.44	1.79	4.29
PA6	0.92	0.31	1.23	5.64
PA7	0.69	0.42	1.11	5.96

**Table 3:** Textural parameters of the biocarbons.

Sample	Total ^1^		Micropore		Pore Size [nm]
	Surface Area [m^2^/g]	Pore Volume [cm^3^/g]	Area [m^2^/g]	Volume [cm^3^/g]	
PA6	320	0.23	276	0.18	3.96
PA7	616	0.48	490	0.40	3.20

^1^ Method error in the range from 2 to 5%.

**Table 4:** The parameters calculated from Langmuir and Freundlich models.

Model	Parameters	Methylene Blue		Methyl Red	
		PA6	PA7	PA6	PA7
	q_e_ [mg/g]	40	146	31	113
Langmuir	q_m_ [mg/g]	41	147	33	115
	R^2^	0.999	0.993	0.990	0.991
	R_L_	0.116–0.316	0.502–0.729	0.512–0.759	0.493–0.722
	K_L_ [L/mg]	0.216	0.016	0.032	0.013
Freundlich	R^2^	0.940	0.990	0.959	0.981
	K_F_ [mg/g(L/mg)^1/n^]	31.20	94.17	13.78	52.88
	1/n	0.132	0.179	0.330	0.293

**Table 5:** Kinetic parameters for adsorption of methylene blue/methyl red.

Model	Parameters	Methylene Blue		Methyl Red	
		PA6	PA7	PA6	PA7
	q_t_ [mg/g]	22	83	31	50
Pseudo-first order	q_e,cal_ [mg/g]	5	14	34	13
	R^2^	0.961	0.949	0.9730	0.885
	k_1_ [1/min]	3.77 × 10^−2^	2.40 × 10^−2^	1.17 × 10^−2^	9.90 × 10^−3^
Pseudo-second order	q_e,cal_ [mg/g]	22	84	33	51
	R^2^	1	0.999	0.994	0.999
	k_2_ [g/mg × min]	2.25 × 10^−2^	2.19 × 10^−3^	9.96 × 10^−4^	2.70 × 10^−3^

**Table 6:** Thermodynamic parameters of the adsorption of methylene blue/methyl red on the biocarbons.

Biocarbon	q_e_ [mg/g]	Temperature [K]	∆G^0^ [kJ/mol]	∆H^0^ [kJ/mol]	∆S^0^ [J/mol K]
PA6 (methylene blue)	37	298	−4.59	9.53	40.11
	40	318	−5.50		
	47	338	−6.49		
PA7 (methylene blue)	80	298	−8.13	10.85	63.61
	98	318	−9.31		
	109	338	−10.69		
PA6 (methyl red)	25	298	−3.15	12.21	51.52
	29	318	−4.17		
	32	338	−5.22		
PA7 (methyl red)	50	298	−8.35	6.88	51.07
	57	318	−9.35		
	63	338	−10.39		

**Table 7:** Methylene blue/methyl red sorption capacity of selected bioadsorbent.

Organic Dye	Bioadsorbent	q_max_ [mg/g]	References
Methylene blue	PA6	41	This study
	PA7	147	This study
	Weeds-based biochar	37	[34]
	Microcrystalline cellulose from oil palm fronds	13	[35]
	Hydrochars from Pinus caribaea	149	[36]
Methyl red	PA6	33	This study
	PA7	115	This study
	Bark of Hopbush	37	[37]
	White potato peel powder	31	[38]
	Coffee residues	77	[39]

## Data Availability

Data is contained within the article.

## Sample Availability

Samples of the compounds are available from the authors.
